# Evolving Treatment Strategies for Elderly Leukemia Patients with IDH Mutations

**DOI:** 10.3390/cancers10060187

**Published:** 2018-06-06

**Authors:** Michael J. Buege, Adam J. DiPippo, Courtney D. DiNardo

**Affiliations:** 1 Pharmacy Clinical Programs, The University of Texas MD Anderson Cancer Center, Houston, TX 77030, USA; mjbuege@mdanderson.org; 2 Department of Leukemia, The University of Texas MD Anderson Cancer Center, Houston, TX 77030, USA; ajdipippo@mdanderson.org

**Keywords:** acute myeloid leukemia, treatment, elderly, enasidenib, AG-221, ivosidenib, AG-120, venetoclax, ABT-199

## Abstract

Acute myeloid leukemia (AML) is a debilitating and life-threatening condition, especially for elderly patients who account for over 50% of diagnoses. For over four decades, standard induction therapy with intensive cytotoxic chemotherapy for AML had remained unchanged. However, for most patients, standard therapy continues to have its shortcomings, especially for elderly patients who may not be able to tolerate the complications from intensive cytotoxic chemotherapy. New research into the development of targeted and alternative therapies has led to a new era in AML therapy. For the nearly 20% of diagnoses harboring a mutation in isocitrate dehydrogenase 1 or 2 (IDH1/2), potential treatment options have undergone a paradigm shift away from intensive cytotoxic chemotherapy and towards targeted therapy alone or in combination with lower intensity chemotherapy. The first FDA approved IDH2 inhibitor was enasidenib in 2017. In addition, IDH1 inhibitors are in ongoing clinical studies, and the oral BCL-2 inhibitor venetoclax shows preliminary efficacy in this subset of patients. These new tools aim to improve outcomes and change the treatment paradigm for elderly patients with IDH mutant AML. However, the challenge of how to best incorporate these agents into standard practice remains.

## 1. Introduction

Acute myeloid leukemia (AML) comprises a heterogeneous group of hematologic malignancies characterized by blastic and abnormally differentiated myeloid clonal populations. It is the most common acute leukemia in adults, with 19,520 new cases (approximately 76% of predicted acute leukemias) and 10,670 deaths estimated to occur in the United States in 2018 [1]. More than 80% of cases are diagnosed at age 45 and above, and more than 50% of diagnoses are made in patients aged 65 years and older [2].

Compared to younger age groups, treatment outcomes for older adults with AML are dismal, particularly for patients aged 75 years and older. Complete remission (CR) rates, which may be predictive of survival, are noted to be lower (40–50%) and of shorter duration in elderly patients [3,4,5,6,7]. While overall survival rates for patients aged 65 to 74 years have improved modestly over the last four decades, they remain unacceptably low with only 30% surviving at one year and less than 10% surviving at five years. Among patients aged 75 and older, one-year survival is less than 15% and has remained stagnant over this time period; patients aged 85 years and older have even demonstrated a slight decline in one-year survival [8].

Reduced survival in this population is multifactorial. Older patients with AML are more likely to have comorbidities impacting ability to receive and tolerate intensive treatment [7,9]. Additionally, AML in older adults appears to be biologically distinct from younger patients, with cytogenetic and molecular abnormalities associated with worse prognosis noted to occur at higher frequencies in this population [10,11,12]. Increasing understanding of distinguishing features of AML in elderly patients has driven efforts to identify more tailored therapies to balance efficacy with expected toxicity, such as the use of hypomethylating agents that are now standard-of-care options for these patients [13,14].

This search for targeted therapies has also included investigations stemming from the longstanding notion that aberrations in cellular metabolism contribute to carcinogenesis [15,16]. Initial genomic analyses of human glioblastoma and related CNS tumors noted recurring mutations in *IDH*, encoding isozymes of isocitrate dehydrogenase that catalyze conversion of isocitrate to α-ketoglutarate (α-KG). Subsequently, whole genome sequencing of patients with AML identified a number of recurring somatic mutations, including *IDH1* [17]. These original discoveries led to a surge of sequencing studies reporting mutations in the IDH1 and IDH2 isozymes in AML and other cancers, and soon after, small molecule inhibitors targeting mutated *IDH1/2* (mIDH) entered the clinic, which have produced exciting results [17,18,19,20,21,22,23,24,25]. In this review, we discuss the role of mIDH in leukemogenesis, and the mechanistic rationale and clinical data for agents targeting AML patient subsets with mIDH. Agents discussed include IDH1/2 inhibitors, hypomethylating agents, and the B-cell lymphoma 2 (BCL-2) inhibitor venetoclax.

## 2. IDH, *R*-2-hydroxyglutarate, and Leukemogenesis

### 2.1. IDH in Normal Cellular Processes

In addition to their canonical roles in oxidative respiration via the mitochondrial tricarboxylic acid (TCA) cycle, isocitrate, α-KG, and reduced nicotinamide adenine dinucleotide phosphate (NADPH) have myriad roles in other physiologic processes [26]. These include participation in extramitochondrial lipid synthesis, cholesterol synthesis, and protection against oxidative cellular insults through antioxidant activity. IDH-mediated catalysis of α-KG and NADPH formation via oxidative decarboxylation of 2*R*,3*S*-isocitrate and NADP^+^ is key to regulation of these metabolic processes.

**Figure 1:**
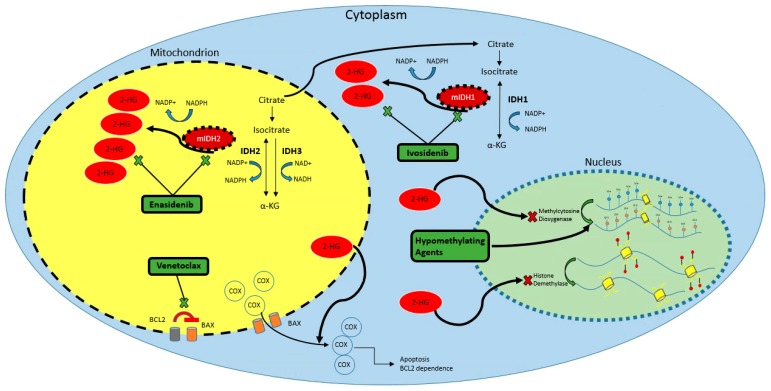
IDH pathway and targets in acute leukemia. IDH1/2 catalyze the conversion of isocitrate to α-KG. However, mutations in the catalytic active site of IDH1/2 causes increased affinity to NADPH and α-KG, leading to accumulation of the oncometabolite 2-HG. 2-HG accumulation has several detrimental effects at the cellular level, including hypermethylation of DNA, silencing in cell differentiation pathways (HOX, MAPK, WNT, TGFβ), and impaired metabolic regulation resulting in apoptosis and BCL2 dependence. Specific inhibitors including enasidenib and ivosidenib bind to mIDH1/2 with a greater affinity than isocitrate allowing normal cellular process to continue and decrease the amount of 2-HG production. Other promising agents work on the downstream effects of 2-HG accumulation, including hypomethylating agents (azacitidine and decitabine) restoring cellular differentiation, as well as venetoclax restoring metabolic regulation and apoptotic pathways. Abbreviations: IDH1 = isocitrate dehydrogenase 1, IDH2 = isocitrate dehydrogenase 2, IDH3 = isocitrate dehydrogenase 3, mIDH1 = mutated IDH1, mIDH2 = mutated IDH2, 2-HG = beta-hydroxyglutarate, α-KG = alpha-ketoglutarate, COX = cytochrome c oxidase, Me = methyl group, OH = hydroxyl group, BCL2 = B-cell lymphoma 2, BAX = BCL2 associated protein X, NADP/H = nicotinamide adenine dinucleotide phosphate, NAD/H = nicotinamide adenine dinucleotide.

The human genome encodes three IDH isozymes [26]. NADP^+^-dependent IDH1 is localized primarily to the cytosol and peroxisomes, while NADP^+^-dependent IDH2 is localized to the mitochondria. IDH3 is also localized to the mitochondria, but unlike IDH1/2, is nicotinamide adenine dinucleotide (NAD^+^)-dependent. When cellular energy conditions necessitate oxidative respiration, IDH2 is active in the traditional TCA cycle, catalyzing dehydrogenation of isocitrate to α-KG and producing NADPH. IDH1 does not participate in the TCA cycle. Importantly, unlike IDH3, IDH1/2 can also participate in reverse reductive carboxylation of isocitrate to α-KG. In energy-rich conditions, IDH3 activity is allosterically suppressed by regulatory co-factors (owing to its extra regulatory subunits), producing accumulation of isocitrate. Some intramitochondrial isocitrate is converted by IDH2 to α-KG, generating NADPH which reduces oxidative damage by reactive oxygen species. Remaining isocitrate is converted by aconitase back to citrate, which is shunted into the cytosol for production of acetyl-CoA and NADPH mediated, in part, by IDH1. [26,27,28,29]. The physiologic and mutant function of IDH1/2, along with the proposed activity of IDH1/2 inhibitors and other targeted therapies, are depicted in Figure 1.

### 2.2. Mutant IDH Function

Recurrent mutations in *IDH1* were identified in 2008 during an integrated genomic analysis of a set of human glioblastoma tumor samples, and subsequently *IDH2* was identified in 2009 in a set of glioma tumor samples [18,19]. Shortly thereafter, recurrent *IDH1/2* mutations were noted in AML in 2009, along with several other solid tumors and myelodysplastic syndrome (MDS) [30,31,32]. The reported frequency of mIDH in AML varies, ranging from 7–14% for *IDH1* and 8–19% for *IDH2*, and mutations of either isozyme have been reported in up to 33% of collective cases [33,34,35,36]. Co-occurring mutations in both isozymes have been reported to be rare, but more recent investigation in a sample of patients with AML, MDS, or chronic myelomonocytic leukemia indicates that simultaneous *IDH1/2* mutations may occur in up to 19% of mIDH patients [37,38,39]. However, one or both genes were detected at low allele frequencies in patients harboring dual mutations and required ultra-deep orthogonal sequencing for confirmation. The clinical significance of co-occurring mIDH remains unknown. Recurrent *IDH1/2* mutations reported in AML are somatic missense mutations affecting highly conserved arginine residues at codon 132 in exon 4 of *IDH1* (IDH1^R132^) and at codons 140 and 172 in exon 4 of *IDH2* (IDH2^R140^ and IDH2^R172^) [17,36]. An additional prognostic germline single-nucleotide polymorphism at codon 105 in exon 4 of *IDH1* has been reported in AML [40,41]. No oncogenic *IDH3* mutations have been reported in AML or other cancers.

Early reports described *IDH1/2* mutations, particularly *IDH1*, as producing loss of function [19,42]. However, *IDH1/2* mutations in AML and other tumors are heterozygous and occur in the active catalytic site, suggesting oncogenic gain of new function rather than loss of tumor suppression [34,43,44]. This appears to be supported by current understanding of the pathophysiologic function of mIDH. In normal cells, the oncometabolite (*R*)-2-hydroxyglutarate (2-HG) is produced at low levels by malate dehydrogenase-mediated reduction of α-KG. Accumulation in normal cells does not occur due to “metabolic proofreading” by endogenous 2-hydroxyglutarate dehydrogenases [45,46]. As described previously, IDH1/2 facilitate both oxidative decarboxylation of isocitrate to α-KG and reverse reductive carboxylation of isocitrate to citrate. Mutant IDH1/2 appear to lose oxidative function as well as normal reductive function, instead demonstrating neomorphic, preferential production of 2-HG at high levels [47,48]. This appears to be due to reduced binding affinity for isocitrate and increased affinity for NADPH, resulting in reduction of α-KG without carboxylation [49]. In addition, hypoxic tumor microenvironments promote glutamine utilization in the TCA cycle, following conversion of glutamine to glutamate and subsequently to α-KG [50,51,52,53]. These conditions appear to contribute to oncogenesis in the presence of mutant *IDH1/2*, as 2-HG in these cells is derived in large proportion from glutamine [52,54].

The metabolic and non-metabolic effects of mIDH-mediated elevations in 2-HG have recently been thoroughly reviewed [55,56]. Accumulating 2-HG competitively inhibits α-KG-dependent dioxygenases, such as the egg-laying-defective nine (EGL-9) prolyl hydroxylases, ten eleven translocation (TET) DNA methylases, and Jumonji C (JmjC) domain-containing histone demethylases, producing leukemogenic impairment of growth regulation and abnormal differentiation through histone and DNA hypermethylation [38,57,58,59,60,61,62]. Hypermethylation appears to silence expression of lactate dehydrogenase (LDH) A, with decreased LDH activity noted in one study of AML patients [63,64]. Reduced LDH function may impair glycolysis, facilitating malignant cell proliferation by providing glucose for pyruvate oxidation and maintenance of the TCA cycle. Increased mitochondrial influx of acetyl-CoA, mediated by increased expression of hypoxia-inducible factor 1α (HIF-1α) and resultant suppressed activity of pyruvate dehydrogenase, may further contribute to TCA activity [55]. This increased dependence on oxidative metabolism also indicates sensitivity to alterations in the electron transport chain (ETC). Cytochrome c oxidase (COX), also known as complex IV, is a component of the ETC, and is known to be inhibited by 2-HG [65,66]. COX inhibition produces a hypoxia-like state, activating the pro-apoptotic proteins BAX and BAK via BCL-2 homology 3 (BH-3) effectors to trigger mitochondrial outer membrane permeabilization (MOMP) leading to apoptosis [67,68,69,70]. Anti-apoptotic BCL-2 antagonizes BAX and BAK, preventing MOMP and promoting cell survival—thus, mutant *IDH* cells display dependence on BCL-2 [68,71,72].

In addition to processes linked to the TCA cycle, mIDH isozymes affect other cellular functions that likely contribute to leukemogenesis. Interestingly, 2-HG accumulation has been linked in vitro to inhibition of the AlkB homolog (ALKBH) DNA repair enzymes, as well as decreased ataxia telangiectasia mutated (*ATM*) expression leading to impaired homologous recombination, both of which may sensitize *IDH*-mutated cells to poly(ADP-ribose) polymerase (PARP) inhibitors [73,74,75]. However, further exploration of this possibility in AML has not yet been reported. CD8^+^ T-cells are also noted to be inhibited by high concentrations of 2-HG, and in vitro models have indicated potential for immune evasion by tumors with mutated *IDH*; this has not yet been demonstrated in AML [76,77,78]. Finally, there is evidence that mutant *IDH* cells’ reduced capacity for producing NADPH may lead to depletions in glutathione, increasing reactive oxygen species and oxidative stress [79,80].

## 3. Targeted Therapies for *IDH*-Mutant AML

### 3.1. Rationale for Combination with Hypomethylating Agents

The increasingly prevalent role of hypomethylating agents (azacitidine, 5-Aza; decitabine, DAC) and growing understanding of mutant *IDH1/2* pathophysiology in AML has recently led to exploration of combined therapy with hypomethylators and mutant *IDH*-targeted therapy. The mechanistic rationale for combining targeted therapy with hypomethylating agents in mIDH AML is described here; clinical data for these combinations and others are described alongside data for monotherapy with each targeted agent below. As previously described, *IDH1/2*-mutated AML displays a hypermethylated DNA phenotype that likely contributes to leukemogenesis [55]. These mutations may serve as initiating events that function in tandem with other somatic genetic lesions frequently present in elderly patients with AML (e.g., *FLT3^ITD^*) to produce epigenetic modifications leading to overt leukemic transformation [11,81,82]. In vitro and in vivo analysis of separate exposure of *Flt3^ITD^;Tet2*-mutant AML monomorphic CD48^+^CD150^−^ multipotent progenitor cells to 5-Aza and the mIDH2 inhibitor enasidenib led to partial normalization of cell differentiation, as well as reversal in many sites of known DNA hypermethylation induced by *Tet2*. However, in vivo RNA sequencing indicated that neither agent alone significantly suppressed the malignant progenitor clone. When both agents were used in combination in this model, hematopoietic differentiation was again noted, along with significant reduction in the proportion of progenitor cells. Combination therapy also produced more potent reversal of both site-specific and global hypermethylation [83]. These results have provided rationale for clinical testing of this combination and others.

### 3.2. Enasidenib

#### 3.2.1. Mechanism

Enasidenib (AG-221, CC-90007, Idhifa^®^; Celgene Corporation, Summit, NJ, USA) is a selective, non-competitive inhibitor of IDH2 recently approved by the United States Food and Drug Administration (FDA) for relapsed/refractory mIDH2 AML. Enasidenib binds to the divalent cation binding helices at the interface of IDH2 homodimers, allosterically maintaining the enzyme in an open conformation to reduce binding affinity for NADPH and impair catalytic activity [84]. Enasidenib demonstrated more than 40-fold increased selectivity for mIDH2 inhibition compared to the wild-type enzyme [85]. Ex vivo preclinical studies in human AML cells treated with enasidenib noted 99% reduction in intracellular 2-HG relative to vehicle-treated controls. At six days following enasidenib treatment, AML cells displayed increased granulosity relative to wild-type samples, with accompanying increased expression of cell-surface markers indicating monocytic and granulocytic differentiation and decreased blast cell percentages, without evidence of inducing early apoptosis. Additional analysis noted reversal of hypermethylation approaching *IDH2* wild-type levels [84,86].

Similar results were obtained in human mIDH2 xenograft and multigenic mouse models, with reduced numbers of IDH2^R140Q^ mutant leukemia cells, increased bone marrow blast differentiation without apoptosis, reduced blood 2-HG levels, reversal of hypermethylation, and increased overall survival [83,86,87,88]. An analysis of samples from a phase I trial in patients with relapsed/refractory AML and either *IDH2^R140^* or *IDH2^R172^* confirmed enasidenib’s potent suppression of 2-HG and normalization of hematopoietic differentiation, including emergence of functional *IDH2*-mutated neutrophils [89].

#### 3.2.2. Clinical Activity

A retrospective cohort analysis of 826 patients, including 167 with *IDH1/2* mutations, was performed for the purpose of establishing an understanding of natural history and prognosis in this population, regardless of treatment regimen [90]. Median age was 62 years. Remission rates, including both CR and CR with incomplete hematologic recovery (CRi) according to AML treatment status were 68% for induction, 42% for first-line salvage therapy, and 27% for second-line and beyond salvage therapy. No differences in response or overall survival (OS) were noted according to *IDH* mutation status, although OS was noted to be 100% at median follow-up of 16 months in a subgroup analysis of patients who were *FLT3^ITD^*-negative, *NPM1*-mutated, and *IDH* wild-type and aged less than 60 years. Median survival for each treatment status was 15.4 months, 8.7 months, and 4.8 months, respectively. This study established that response rate and survival for mIDH and wild-type patients were similar in an older patient population.

Clinical trial outcomes for enasidenib-based therapy in *IDH*-mutated AML, as well as ongoing clinical trials, are summarized in Table 1. A first-in-human phase 1/2 dose escalation study was performed to assess use of enasidenib in patients with relapsed/refractory *IDH2*-mutated AML, with results from the phase 1 dose escalation (113 patients) and expansion (126 patients) phases reported [91]. Median age was 67 years, ranging up to 100 years. The maximum tolerated dose was not reached at daily doses up to 650 mg. Enasidenib 100 mg daily in continuous 28-day cycles was selected for the expansion phase based on demonstrated efficacy, maximal 2-HG suppression at doses ≥ 100 mg, and the lowest proportion of patients whose dose was reduced for toxicity relative to higher dose levels [85,91]. In this heavily pretreated population (32% refractory to initial induction or re-induction, 23% relapsed/refractory to at least two cycles of first-line lower-intensity therapy), overall response rate (ORR) was 40% including 19% achieving CR, and median duration of response was 5.8 months. Median OS was 9.3 months; in patients achieving CR, median OS was 19.7 months and median duration of response was not reached. Of note, median time to CR was 3.7 months, ranging from 0.7 to 11.2 months. The most common adverse events were indirect hyperbilirubinemia (38%) and nausea (23%). Grade 3 or 4 adverse events included hyperbilirubinemia (12%), differentiation syndrome (DS, 6%), and tumor lysis syndrome (TLS, 3%); rates of grade 3 or 4 hematologic adverse events were less than 10%. The response rate in this trial and clinically meaningful duration of response and survival led to regular FDA approval of enasidenib 100 mg daily as monotherapy for *IDH2*-mutated AML in the relapsed/refractory setting. 

Of note, a later analysis of patients in the above trial who maintained stable disease (SD) through day 90 of treatment (89 patients, 42%) noted that 27% of these patients experienced “late response” at a median of 130 days, while 45% maintained SD to a median of 173 days and 28% progressed [105]. This demonstrated that SD is not necessarily an indicator of treatment failure in this setting, and consideration should be given to continuing treatment until progression or unacceptable toxicity. Enasidenib is being further evaluated in the relapsed/refractory AML setting in the phase 3 randomized IDHENTIFY trial, in comparison to best supportive care, 5-Aza, or low- or intermediate-dose cytarabine [98].

Enasidenib has also been evaluated in the front-line setting for older patients who were not candidates for standard therapy. Patients (*n* = 37) with untreated *IDH2*-mutated AML who were aged 60 or older (median 77 years) were enrolled within the original AG221-001 phase 1/2 dose escalation study, with the dose expansion phase utilizing the 100 mg daily dose in continuous 28-day cycles. ORR was 37.8%, with 19% achieving CR at a median time of 5.6 months ranging up to 12.9 months. Median duration of response was 12.2 months; median duration of CR was not reached. Median OS for responders and non-responders was 19.8 months and 5.4 months, respectively. The most common adverse events were fatigue (43%), nausea (41%), and decreased appetite (41%). DS was reported in 8%, and TLS was reported in 5% [93].

Additional ongoing studies are evaluating front-line regimens in combination with enasidenib. A phase 1 trial of patients with newly diagnosed *IDH*-mutated AML is utilizing standard 7 + 3 chemotherapy (anthracycline for three days and continuous infusion of cytarabine for seven days) in combination with ivosidenib 500 mg daily or enasidenib 100 mg daily for mutant *IDH1* or *IDH2*, respectively [94]. Preliminary analysis of 65 patients (38 receiving enasidenib) noted CR, CRi, or complete response with incomplete platelet recovery (CRp) in 67% of enasidenib patients with de novo AML and 50% of patients with secondary AML. Eight enasidenib-treated patients proceeded to stem cell transplant. The most frequent grade ≥3 non-hematologic adverse events were febrile neutropenia (63%), hypertension (11%), colitis (8%), and maculopapular rash (8%). A phase 1/2 trial of patients with newly diagnosed *IDH*-mutated AML who are ineligible for intensive induction therapy is utilizing 5-Aza in combination with ivosidenib 500 mg daily or enasidenib 100 mg or 200 mg daily for patients with *IDH1* or *IDH2* mutations, respectively [96]. Preliminary phase 1b results for six patients receiving enasidenib with 5-Aza (median age 68 years) noted responses in three (50%) patients.

#### 3.2.3. Safety and Tolerability—General

The FDA’s review of safety data for enasidenib utilized a pooled safety population of 214 patients with relapsed/refractory AML, all of whom received at least one 100 mg dose of enasidenib [85]. Among these patients, 73% received enasidenib 100 mg daily for more than three months. The adverse events suspected to be associated with enasidenib use are summarized in Table 2. Treatment-emergent serious adverse events were reported in 78% of patients. The most commonly reported adverse events associated with enasidenib were hyperbilirubinemia (up to 83%) rarely associated with concomitant transaminitis (≤3%), and nausea (28%). In the FDA report, hyperbilirubinemia of any grade was reported by the investigator in 32% of patients. The FDA safety review identified bilirubin elevations in 83% of patients from the pooled safety data. Notably, enasidenib is known to inhibit UDP glucuronosyltransferase 1A1 (UGT1A1), the enzyme principally responsible for bilirubin metabolism [91]. Thus, hyperbilirubinemia is primarily due to indirect bilirubin elevations in this setting, and enasidenib does not appear to be associated with direct liver toxicity. Treatment interruption, dose reduction, or discontinuation due to adverse events occurred in 53%, 10%, and 11% of patients, respectively. No specific adverse event accounted for a distinguishing number of treatment interruptions, dose reductions, or discontinuations. There were 127 deaths reported (59%), 62 (29%) of which occurred while receiving enasidenib. Death narratives were available for 109 of these patients; only one of these patients’ deaths was considered possibly related to enasidenib.

Enasidenib’s approved labeling contains a statement indicating a possible risk of TLS [87]. This is based on TLS occurrence in 13 patients (6%), most of which were grade 3, and one of which was fatal. However, upon review of available event narratives and clinical data, the FDA noted that nearly all TLS events occurred in the presence of rising white blood cell counts [85]. Based on this information and enasidenib’s mechanism, it was concluded that instances of TLS were likely to be related to underlying malignancy, and that patients receiving enasidenib do not appear to be at increased risk of TLS relative to other patients with AML. 

#### 3.2.4. Differentiation Syndrome

Originally dubbed retinoic acid syndrome, DS was first described with the use of the differentiating agents all-*trans* retinoic acid and arsenic trioxide for acute promyelocytic leukemia (APL) [106]. These agents produce degradation of the promyelocytic leukemia-retinoic acid receptor alpha fusion protein, allowing for normal myeloid differentiation [107]. The pathophysiology of DS is incompletely understood. Proposed mechanisms include stimulation of chemokine production by pulmonary alveoli and overproduction of inflammatory cytokines and adhesion molecules by differentiating cells, leading to widespread infiltration of myeloid cells and leukocytes into organ tissues [108]. Clinical presentation of APL treatment-associated DS is nonspecific and most commonly includes hyperleukocytosis, dyspnea, respiratory distress, pulmonary edema or infiltrates, pleural or pericardial effusion, and fever. Less common presentations include weight gain, bone pain, headache, hypotension, congestive heart failure, acute renal failure, and hepatotoxicity. Typically, onset of APL treatment-associated DS is within 7–12 days of treatment initiation, and it has been reported to occur in 26–31% of patients [109,110,111]. Corticosteroids are the mainstay of APL treatment-associated DS management; as alveolar chemokine secretion appears to be an initiating event, declining respiratory function may be considered an early sign of DS and indication to initiate steroid therapy (e.g., dexamethasone 10 mg twice daily until symptom resolution, followed by a two-week taper) [108,112].

Enasidenib-induced DS was first noted in the initial phase 1 clinical trial in relapsed/refractory AML, approximately 1 month after study initiation [85,91]. An independent differentiation syndrome review committee (DSRC) was established by the study investigators to retrospectively review possible cases of enasidenib-induced DS [113]. The DSRC criteria for DS events included evidence of differentiation in peripheral blood counts or rapid response to steroid therapy. Two hundred eighty-one patients had been enrolled at the time of the DSRC report. Signs and symptoms of enasidenib-induced DS appeared to be similar to those noted in APL treatment-induced DS. However, in contrast to APL treatment-associated DS, 11.7% of patients were identified as having experienced possible or probable DS. Additionally, the median onset of enasidenib-induced DS was 30 days, ranging from 7–129 days. Among the 33 patients with possible or probable DS, 28 (85%) received steroid therapy, and 46% had enasidenib interrupted until symptom resolution. Two patients (6%) were dose-reduced; none required permanent discontinuation of enasidenib because of DS. The authors noted that patients who experienced DS were significantly less likely to have less than 20% bone marrow blasts (median 6% vs. 22%) and were more likely to have received fewer previous lines of AML therapy (median 1 vs. 2 lines).

Management was similar to APL treatment-induced DS [113]. Dexamethasone 10 mg twice daily was initiated, along with empiric therapy for other possible etiologies (e.g., infection). In cases of hyperleukocytosis, hydroxyurea was used for leukoreduction. However, the FDA safety review concluded that enasidenib does not appear to induce hyperleukocytosis, and that these events likely represent progression of underlying malignancy [85]. As DS may be life-threatening, early recognition and evaluation of signs and symptoms is crucial.

In addition to the DSRC report, the above-described FDA safety review included an independent review of pooled safety data to identify possible DS events [85]. The FDA review included any events identified by the DSRC, as well as patients identified using an algorithm which included effusion or pulmonary edema as qualifying events for possible DS, along with several other criteria. Seventy-eight patients (36%) were identified by the FDA algorithm as having experienced possible or probable DS events. FDA’s conclusion was DS incidence is likely between 13% (rate reported by enasidenib’s manufacturer prior to publication of the final DSRC report) and 33% (rate identified by the FDA’s algorithm, excluding events identified exclusively by the DSRC). Based on this information, the FDA’s reviewer recommended a boxed warning for DS which was incorporated into enasidenib’s labeling [87].

### 3.3. Ivosidenib

#### 3.3.1. Mechanism

Ivosidenib (AG-120) is a selective, reversible, potent inhibitor of mutant IDH1 currently undergoing phase 1–3 trials for treatment of newly diagnosed *IDH1*-mutated AML [99,101]. Exposure of primary human AML cells expressing mutant IDH1, as well as cells cultured ex vivo, to ivosidenib reduced intracellular 2-HG concentrations and induced myeloid differentiation. Additionally, in TF-1 mutant *IDH1^R132H^* cells, treatment with ivosidenib restored erythropoietin-induced differentiation [114]. In a sample of patients with *IDH1*-mutated advanced hematologic malignancies, plasma 2-HG levels were reduced by up to 99.7% after multiple doses of ivosidenib and were similar to those observed in healthy volunteers. Similar reduction was noted in bone marrow (up to 99.9%) [115].

#### 3.3.2. Clinical Activity

Clinical trial outcomes for ivosidenib-based therapy in *IDH*-mutated AML, as well as ongoing clinical trials, are summarized in Table 1. The first-in-human use of ivosidenib was reported in an ongoing phase 1 trial of patients with *IDH1*-mutated hematologic malignancies, including both treatment-naïve and relapsed/refractory AML [100]. Preliminary results for patients with relapsed/refractory disease receiving ivosidenib 500 mg daily (*n* = 125) noted an ORR of 42%, with CR/CRi in 30% of patients for a median duration of 8.2 months. Among all patients enrolled (*n* = 258), the most common all-grade adverse events were diarrhea (33%), leukocytosis (30%), nausea (30%), fatigue (29%), febrile neutropenia (25%), dyspnea (24%), anemia (23%), QT prolongation (23%), peripheral edema (22%), pyrexia (21%), and decreased appetite (20%). All-grade and grade ≥ 3 DS was noted in 11% and 5% of patients, respectively [99].

Bone marrow mononuclear cells from patients in another phase I study of ivosidenib in advanced hematologic malignancies were assessed for mutant *IDH1* variant allele frequency to assess the depth of mIDH1 clearance [116]. At the time of reporting, clearance (defined as mIDH1 frequency below the limit of detection, ≤0.04%) was noted in six of 25 (24%) patients with relapsed/refractory AML and 3 of 5 (60%) patients with untreated AML who achieved CR, as well as two of four (50%) patients with untreated AML who achieved CRi. Mutant *IDH1* clearance did not occur in any patients who did not achieve best response of CR or CRi. However, the clinical impact of mIDH1 clearance requires further study. While formal studies of ivosidenib-induced hematopoietic cell differentiation using clinical samples have not yet been reported, ivosidenib-induced DS has been reported in a three-patient case series [117].

Additional clinical trials of ivosidenib as part of combination therapy are ongoing. In the above-described phase 1 front-line trial of enasidenib or ivosidenib in combination with standard 7 + 3 chemotherapy, 27 patients have been treated with ivosidenib (median age 60 years), and 23 were evaluable for response at the time of publication [94]. Collectively, CR/CRi/CRp occurred in 86% of patients with de novo AML and 44% of patients with secondary AML. Six ivosidenib-treated patients proceeded to stem cell transplant. The most common adverse events were febrile neutropenia (56%), transaminitis (11%), and colitis (11%). The aforementioned phase 1/2 trial of enasidenib or ivosidenib in combination with 5-Aza in patients with newly diagnosed AML who are ineligible for intensive chemotherapy reported 11 patients (median age 81 years) receiving ivosidenib in the phase 1b component [96]. Response was noted in eight patients, four achieved CR, one patient achieved CRi, one patient achieved PR, two patients had no morphologic evidence of leukemia (MLFS), and three patients maintained SD. Finally, ivosidenib is being tested in an ongoing phase 3, front-line, randomized, placebo-controlled trial in combination with 5-Aza versus 5-Aza alone [101].

### 3.4. Venetoclax

#### 3.4.1. Mechanism and Clinical Activity

As described previously, 2-HG suppresses COX and leads to dependence of AML cells on BCL-2 for continued viability [71]. Venetoclax (ABT-199, GDC-0199, Venclexta^®^; AbbVie Inc., North Chicago, IL, USA) is a highly selective oral agent that binds the BH-3 binding groove of BCL-2, preventing BCL-2-mediated inactivation of pro-apoptotic proteins [118,119]. Importantly, venetoclax does not bind BCL-extra large (BCL-xL), avoiding off-target induction of platelet apoptosis seen with other investigational BCL-2 inhibitors (e.g., obatoclax). Venetoclax is currently FDA-approved for use in chronic lymphocytic leukemia (CLL) [120]. While not directly targeting mIDH, venetoclax has also shown promising results as part of combination therapy for older adults with AML, including particular sensitivity in mIDH subsets [121].

Clinical trial outcomes for venetoclax-based therapy in *IDH*-mutated AML, as well as ongoing clinical trials, are summarized in Table 1. In a phase 2 study of venetoclax 800 mg daily in patients with relapsed/refractory AML or unable to receive intensive chemotherapy, *IDH1/2* mutations were detected in 12 (38%) patients [102]. Among these, four (33%) achieved CR/CRi. Additionally, a recently reported phase 1b dose escalation study of venetoclax in combination with 5-Aza or DAC enrolled 17 patients with *IDH1/2* mutations [103]. Among these patients, 10 (59%) demonstrated CR/CRi and three (18%) demonstrated a morphologic leukemia-free state [103]. In a retrospective review of off-label use of venetoclax in combination with 5-Aza, DAC, or low-dose cytarabine, eleven patients had *IDH1/2* mutations [122]. Among these patients, three (27%) had a response, including CR, CRi, and MLFS, in one patient each. Additionally, one patient experienced >50% bone marrow blast reduction without peripheral count recovery, and one patient cleared all peripheral blasts within the first two weeks of treatment but discontinued treatment due to infectious complications.

#### 3.4.2. Safety and Tolerability

It is important to note that the venetoclax doses used in the small *IDH*-mutated AML studies described above were frequently higher (ranging from 400 mg to 1200 mg daily) than the FDA-approved CLL dose of 400 mg daily [102,103,122]. The most frequently reported adverse events with venetoclax monotherapy in the above-described phase 2 study in relapsed/refractory AML were nausea, vomiting, diarrhea, febrile neutropenia, and hypokalemia. However, additional data from larger samples with more established dosing are needed to better characterize toxicity with venetoclax monotherapy or combination strategies in this population.

Additionally important to note is the risk of TLS associated with venetoclax. TLS is a well-recognized phenomenon caused by deposition of malignant cellular components into the bloodstream following lysis, manifesting as rapidly evolving electrolyte derangements with possible life-threatening clinical sequelae [123,124]. In the first-in-human study of venetoclax, 10 patients with CLL experienced TLS—three of these patients had clinical TLS at venetoclax doses ranging from 50 mg to 1200 mg, all of whom died as a result of these complications [125]. This led to protocol amendments incorporating a more conservative dose-ramping regimen as well as subsequent studies characterizing risk stratification and mitigation strategies for venetoclax-induced TLS [126]. A recently presented pooled analysis of three studies of venetoclax in CLL noted four cases of laboratory TLS with no clinical TLS, none of which required treatment discontinuation [127]. No TLS was noted in the phase 1b or phase 2 studies for patients with AML described above—incidence was not described in the retrospective review, but TLS prophylaxis was administered [102,103,128]. The strategies established for TLS risk management with venetoclax in patients with CLL provide a framework for safe use in *IDH*-mutated AML. Additionally, TLS risk with venetoclax is likely to be highest in treatment-naïve patients, those with high disease burden, and high-risk disease (e.g., CLL) [126]. Thus, as evidenced by the above-described early clinical trials, risk of venetoclax-induced TLS may be lower for patients with mIDH AML. However, further study and continued vigilance on the part of treating clinicians are needed to ensure patient safety.

The phase 1b study described above also included a cohort of patients receiving venetoclax and DAC alongside the triazole antifungal posaconazole—a well-known inhibitor of cytochrome P450 isozyme 3A4 (CYP3A4)—to assess posaconazole’s effects on venetoclax pharmacokinetics [103]. Venetoclax is primarily metabolized by CYP3A4 and 3A5, and co-administration with strong CYP3A4 inhibitors such as triazole antifungals increases exposure to venetoclax up to 6.4-fold [129]. This led to the inclusion of recommendations for venetoclax dose reductions of at least 50% or 75% in the presence of concomitant moderate or strong CYP3A4 inhibitors, respectively, in the product labeling [130]. As a large majority of AML patients can be expected to receive triazole antifungal prophylaxis, it is important that these interactions be taken into consideration as venetoclax use increases in AML populations [131].

## 4. Conclusions and Future Directions

Based on available evidence, patients with mIDH AML appear to have improved response and survival with the use of single-agent oral mutant IDH targeted agents compared to standard therapies in the relapsed setting. The magnitude of improvement and duration of response, as well as evaluation of long-term toxicities, will be further quantified and better elucidated with time. Furthermore, as enasidenib is the first targeted therapy approved for mIDH AML, it is not yet clear how to best incorporate this and other targeted agents into routine clinical practice. It is likely that combination approaches, i.e., with hypomethylating agent therapy or with other oral small molecule inhibitors, will soon become the new standard. Ongoing studies of targeted agents with hypomethylating agents and other combinations will help to answer these questions and may reshape the treatment landscape of elderly patients with AML. As balancing efficacy and toxicity is of special importance in this patient population, this reshaping may take the form of a treatment approach resembling outpatient chronic disease state management.

Therapy for AML saw a leap forward in 2017, with the FDA approval of enasidenib and renewed enthusiasm for targeted agents, including IDH1/2 inhibitors and venetoclax. These agents appear to complement and—particularly in elderly patients—may reduce or eliminate use of intensive cytotoxic chemotherapy. It is important to note that this may necessitate a paradigm shift in the approach to AML induction therapy away from quickly achieving CR in elderly patients. Given the promising results of early trials, such a shift may bear significant long-term benefits for this population.

## Figures and Tables

**Table 1:** Clinical trials for targeted therapies in patients with mIDH AML **^a^**.

Phase ^b^	Treatment Setting ^c^	Intervention	No. of Patients ^d^	Age, y	ORR, % ^e^	Combined CR/CRi/CRp, %	Time to CR, mo	OS, mo (Median, 95% CI) ^f^	Reference
Enasidenib									
1/2	R/R	Enasidenib monotherapy	109	67 (19–100)	38.5	26.6	3.7 (0.7–11.2)	9.3	[91,92]
	Front-line		37	77 (58–87)	37.8	18.9	5.6 (3.4–12.9)	10.4 (5.7–15.1)	[92,93]
1	Front-line	Enasidenib plus 7 + 3 ^g^	38	63 (32–76)	81	62	NR ^h^	NR	[94,95]
1/2	Front-line	Enasidenib plus 5-Aza	6	68 (64–79)	50	33.3	NR	NR	[96,97]
3	R/R	Enasidenib monotherapy vs BSC ^i^, 5-Aza, or cytarabine	No results available						[98]
Ivosidenib									
1	R/R	Ivosidenib monotherapy	125	NR	41.6	30.4	NR	NR	[99,100]
1	Front-line	Ivosidenib plus 7 + 3	27	60 (24–76)	83	70	NR	NR	[94,95]
1/2	Front-line	Ivosidenib plus 5-Aza	5	81 (72–88)	60	60	NR	NR	[96,97]
3	Front-line	Ivosidenib plus 5-Aza vs. 5-Aza plus placebo	No results available						[101]
Venetoclax									
2 ^j^	R/R	Venetoclax monotherapy	12	71 (19–84) ^k^	NR	33	NR	NR	[102]
1	Front-line	Venetoclax plus 5-Aza or DAC	17	≥65 ^l^	77	59	NR	NR	[103,104]

^a^ Median (range), unless noted otherwise; ^b^ Trials ongoing unless noted otherwise; ^c^ R/R: Relapsed/refractory; ^d^ Response-evaluable with mIDH; ^e^ CR, CRi, complete response with incomplete platelet recovery (CRp), partial remission, or morphologic leukemia-free state; ^f^ OS: Overall survival; CI: Confidence interval; ^g^ 7 + 3: anthracycline for three days and continuous infusion of cytarabine for seven days; ^h^ BSC: Best supportive care; ^i^ NR: Not reported; ^j^ Completed; ^k^ Includes non-mIDH patients; ^l^ Median age not reported.

**Table 2:** Enasidenib-related treatment-emergent adverse events ^a^ [85].

Adverse Event	Any Grade, %	Grade ≥ 3, %
Hyperbilirubinemia ^b^	32–83	7–15
Differentiation syndrome ^b^	13–33	7–17
Nausea	28	2
Decreased appetite	19	2
Fatigue	18	2
Vomiting	17	1
Diarrhea	16	1
Hepatic injury ^c^	14	3
Rash	13	2
Dysgeusia	10	0
Dyspnea ^c^	10	6
Leukocytosis	7	2
Peripheral neuropathy	7	0
Anemia	7	6
Pyrexia	7	1
Hyperuricemia	6	1
Renal insufficiency	5	1
Weight decrease	5	0
Edema ^c^	5	1

^a^ From FDA safety review of pooled data for patients receiving enasidenib 100 mg daily (*n* = 214). ^b^ Lower limit represents investigator-reported rate; upper limit represents FDA audit-reported rate. ^c^ May be related to differentiation syndrome.
